# Bruceine E, a natural quassinoid from Brucea javanica, inhibits PARthanatos via targeting PARP1 in ischemic stroke

**DOI:** 10.3389/fphar.2026.1843673

**Published:** 2026-06-22

**Authors:** Chang Wei, Chun-ze Zhai, Chang-ling Yue, Yi-zhe Wang, Yin Cao, Qian-wen Yang, Zhao-huan Zhang, Xiao-hui Xu

**Affiliations:** 1 School of Basic Medical Sciences, Wannan Medical University, Wuhu, China; 2 Anhui Province Key Laboratory of Basic Research and Transformation of Age-related Diseases,Wannan Medical University, Wuhu, China; 3 Department of Laboratory Medicine, Changzheng Hospital, Naval Medical University, Shanghai, China

**Keywords:** bruceine E, ischemic stroke, PARP1 inhibitor, parthanatos, traditional Chinese medicine monomer library

## Abstract

Ischemic stroke currently lacks evidence-based neuroprotective agents, primarily due to the challenge of timely intervention, which often occurs after the onset of irreversible neuronal damage. To address this, this study investigates the PARthanatos pathway, a form of regulated cell death triggered by DNA damage. Utilizing MNNG-induced cellular PARthanatos models, we screened a library of 2,939 traditional Chinese medicine monomers and identified Bruceine E, a natural product derived from Brucea javanica (bitterwood), as a potent inhibitor of PARthanatos at nanomolar concentrations, acting via the inhibition of PARP-1 overactivation. Bruceine E effectively prevents the accumulation of PAR-modified proteins, mitigates mitochondrial membrane potential collapse, and inhibits AIF nuclear translocation. Mechanistically, molecular docking, molecular dynamics simulations, surface plasmon resonance (SPR), and thermal stability assays demonstrate that Bruceine E interacts with the NAD + catalytic pocket of PARP-1 through six hydrogen bonds, exhibiting fast-binding and slow-dissociation kinetics. Furthermore, PARP1 overexpression rescue experiments confirmed that PARP1 overexpression markedly reversed the neuroprotective effect of Bruceine E, indicating that its pharmacological action is specifically dependent on PARP1 regulation. In a permanent distal middle cerebral artery occlusion (pdMCAO) model of C57BL/6 mice, a single intraperitoneal injection of 10 mg/kg Bruceine E administered 4.5 h after occlusion reduced infarct volume by approximately 80.2%, histological evidence confirmed that a single intraperitoneal administration of BE provided effective neuroprotection against ischemic brain injury.

## Introduction

Ischemic stroke, also known as cerebral ischemic stroke (CIS), represents a significant threat to global health, ranking among the leading causes of mortality. According to the 2025 World Stroke Organization (WSO) Global Stroke Fact Sheet, ischemic stroke accounts for over 65% of all stroke cases worldwide, with approximately 7.8 million new incident cases reported annually. In China, the latest 2025 epidemiological data indicate around 2.77 million new cases each year, and its age-standardized incidence rate is markedly higher than the global average (Li et al., 2024; Feigin et al., 2025; Wang et al., 2025). Alarmingly, emerging evidence suggests a younger-onset trend in ischemic stroke: more than 12% of global cases now occur in individuals aged 15–49 years, reflecting a rising disease burden among younger populations (Feigin et al., 2025). The pathophysiology of ischemic stroke involves rapid neuronal loss, with approximately 1.9 million neurons lost per minute following the onset of the event, and irreversible brain tissue damage typically occurring within 6 hours of onset (Saver, 2006). Despite advances in treatment, such as vascular recanalization, more than 50% of patients experience significant disability post-intervention. Correspondingly, in 2021,ischemic stroke accounted for a total of 70.4 million disability-adjusted life years (DALYs) lost globally (Pu et al., 2023), representing over 3% of the total disease burden (Li et al., 2024).

This condition imposes substantial challenges on families, healthcare systems, and society at large. Despite the considerable burden, therapeutic options remain limited. While intravenous thrombolysis and mechanical thrombectomy are effective in recanalizing occluded vessels, their efficacy is constrained by a narrow therapeutic window of 4.5 h. Moreover, reperfusion injury and hemorrhagic transformation present significant challenges, and ultimately, less than 20% of patients with ischemic stroke benefit from these interventions (Wang et al., 2022). A more pressing issue is the absence of an evidence-based and clinically available neuroprotective drug that demonstrably improves patient prognosis (Lyden, 2026). Since the 1970s, researchers have explored over 1,000 candidate molecules, including the free radical scavenger NXY-059, magnesium, calcium antagonists, and excitotoxicity inhibitors such as NA-1 and the GABA modulator S44819. Despite promising results in animal models, these candidates have consistently failed in phase III clinical trials (Chamorro et al., 2016; Lyden, 2026). The reasons for these failures are multifaceted, including significant discrepancies between animal models and human patients (e.g., young, healthy male rats versus elderly patients with comorbidities), the absence of a reperfusion context in trial designs and methodological shortcomings such as insufficient sample sizes, low statistical power, and a lack of subgroup analyses. Of note, in some trials, over 37% of subjects did not receive thrombolysis or thrombectomy, which would prevent systemically administered drugs from reaching the lesion site via blood flow (Chamorro et al., 2016; Aksenov and Doubovikov, 2025). Fundamentally, the core issue remains our inability to effectively target the “irretrievable” link within the ischemic cascade.

In recent years, advancements in cell death biology have brought attention to a previously overlooked phenomenon known as PARthanatos. Unlike classical forms of cell death such as apoptosis, necrosis, or pyroptosis, PARthanatos is initiated by the overactivation of poly (ADP-ribose) polymerase-1 (PARP-1) in response to DNA single-strand breaks. This process results in the accumulation of PAR polymers, increased mitochondrial membrane permeability, translocation of apoptosis-inducing factor (AIF) to the nucleus, and ultimately, leads to extensive DNA fragmentation and energy depletion (Koehler et al., 2021). Apoptosis is an energy-dependent programmed death characterized by caspase activation, cell shrinkage, and apoptotic body formation with minimal inflammation; Necrosis results in plasma membrane breakdown, generalized swelling of the cytoplasm and organelles, moderate chromatin condensation, and spillage of cellular constituents into the microenvironment, triggering a strong pro-inflammatory response (Qi et al., 2025) PARthanatos possesses a distinct identity beyond both: programmed initiation yet suicidal energy depletion. PARP-1 hyperactivation catastrophically consumes NAD^+^/ATP—distinct from the ATP-conserving nature of apoptosis and the passive energy failure of necrosis; it executes death via the PAR-AIF pathway independent of caspases, and although terminal membrane rupture resembles necrosis, it is strictly regulated by PARP-1 (Huang et al., 2022; Yang et al., 2024). Consequently, it is considered the “first wave” of cell death following ischemic events (Liu et al., 2022). Notably, PARthanatos cannot be inhibited by blockers of other cell death pathways once initiated, underscoring its critical role in neuronal death following ischemic stroke (Yue et al., 2025). Studies utilizing transgenic mouse models have demonstrated that deletion of PARP-1 or mutation of the AIF nuclear localization sequence can significantly reduce infarct volume by 40%–60%, without impacting cerebral blood flow, indicating the potential therapeutic value of targeting the PARthanatos pathway (Liu et al., 2022). Unfortunately, currently clinically used PARP inhibitors such as olaparib have significant systemic toxicity and poor blood-brain barrier (BBB) penetration, and no trials have been conducted in the stroke field (Koehler et al., 2021).

Traditional Chinese medicine, refined through millennia of clinical practice, offers distinct advantages due to its multiple bioactive components multifaceted components, diverse targets, and low toxicity, positioning it as a valuable resource for the discovery of novel small molecule drugs. Contemporary pharmacological research has demonstrated that compounds such as quercetin (Bahar et al., 2017), resveratrol (Koshkina et al., 2024), silymarin (Bai et al., 2018), and flavonoids (Fatokun et al., 2013) can traverse the blood-brain barrier, indirectly inhibit PARP-1 enzyme activity, and reduce PAR polymer levels at micromolar concentrations. Nonetheless, these findings primarily focus on PAR polymer accumulation, lacking comprehensive screening and evaluation concerning the “PARthanatos” signaling pathway. The precise sites of action, structure-activity relationships, pharmacodynamic windows *in vivo*, and safety profiles remain inadequately elucidated. Consequently, we propose to screen a library of traditional Chinese medicine monomers using the MNNG-treated PC-9 cell line, a cellular model of PARthanatos, to identify novel, effective inhibitors of PARthanatos.Through this screening, we identified Bruceine E, a quassinoid compound derived from Brucea javanica, as a potent blocker of PARthanatos progression. Bruceine E has been previously reported to exhibit hepatoprotective effects against non-alcoholic fatty liver disease and hypoglycemic activity in diabetic models (Ablat et al., 2017; Man et al., 2025). Notably, both conditions are characterized by impaired mitochondrial bioenergetics and elevated oxidative stress—pathological features that parallel the NAD^+^/ATP depletion and reactive oxygen species generation central to PARthanatos execution (Murata et al., 2019; Dong et al., 2024; Yu et al., 2024; Fang et al., 2025). These metabolic protective properties suggest that Bruceine E may possess intrinsic mitochondrial-protective and energy-modulating capacities, rendering its neuroprotective potential against PARthanatos-driven neuronal death mechanistically plausible. However, whether Bruceine E can directly modulate the PARP-1–AIF axis or protect against ischemic brain injury via PARthanatos inhibition remains entirely unexplored. Given the validated anti-inflammatory and antioxidant capacities of Bruceine E, we hypothesized that this screened natural monomer might suppress excessive PARP1 activation, abrogate neuronal PARthanatos, and ultimately ameliorate cerebral ischemic damage to achieve neuroprotection. To validate this hypothesis and fill the aforementioned research gap, the present study systematically investigated the neuroprotective potential and underlying molecular mechanisms of Bruceine E. Mechanistic and pharmacological validations were performed using an MNNG-induced PARthanatos model in PC-9 cells and a murine permanent cerebral ischemia model, aiming to verify the inhibitory effect of Bruceine E on the PARthanatos signaling pathway and confirm its feasibility as a promising novel candidate for ischemic stroke intervention.

## Materials and methods

All antibodies used in the experiments are list in Supplementary Table 1. All chemicals used in the experiments are list in Supplementary Table 2.

### Materials

Bruceine E (CAS: 21586-90-3, purity: 99.88%, MW:412.43 Da, Chemical Formula: C20H28O9), 13,21-Dihydroeurycomanone (CAS: 129587-06-0, purity: 98.01%, 410.42,C_20_H_26_O_9_), Bruceine D (CAS: 21499-66-1, purity: 99.99%, MW:410.42 Da, Chemical Formula: C20H26O9), Brusatol (CAS: 14907-98-3, purity: 99.95%, MW: 520.53 Da, Chemical Formula: C26H32O11), Yadanziolide A (CAS: 95258-14-3, purity: 99.54%, MW: 426.41 Da, Chemical Formula: C20H26O10) and Pasakbumin B (CAS: 138809-10-6, purity: 99.93%, MW: 424.4 Da, Chemical Formula: C20H24O10) was purchased from TargetMol. Olaparib (OLA; CAS: 763113-22-0, purity: 99.98%, MW:434.46, Chemical Formula: C_24_H_23_FN_4_O_3_) and Methylnitronitrosoguanidine (MNNG; CAS: 70-25-7, purity:99.97%, MW: 147.09, Chemical Formula: C2H5N5O3) was purchased from MedChemExpress (MCE). SH-SY5Y (RRID:CVCL_0019) and PC-9 cells (RRID:CVCL_B260) were purchased from the Chinese Academy of Sciences Cell Bank (Shanghai, China). All compounds were obtained from MCE (Shanghai, China) or TargetMol.

### Primary cortical neuron culture

Following previously established protocols (Wang et al., 2016) Primary cortical neurons were isolated from embryonic day 18.5 (E18.5) C57BL/6 mouse embryos. Under sterile conditions, the bilateral dorsolateral cortex of mouse embryos was rapidly dissected in ice-cold calcium- and magnesium-free Hanks’ balanced salt solution (HBSS). After careful removal of the meninges, blood vessels, and contaminating tissues such as the hippocampus and striatum, the cortical tissue was thoroughly minced. The minced tissue was then transferred to pre-warmed (37 °C) calcium- and magnesium-free HBSS containing an equal volume of 0.25% trypsin, yielding a final trypsin concentration of 0.125%. Digestion was performed at 37 °C for 15 min. The enzymatic reaction was terminated by adding culture medium supplemented with 20% fetal bovine serum. The tissue was gently and repeatedly triturated to obtain a single-cell suspension, which was then filtered through a cell strainer to remove undigested tissue fragments. After centrifugation, the cells were resuspended in DMEM containing 20% fetal bovine serum and seeded into 12-well plates pre-coated with poly-L-lysine overnight at 4 °C. Cells were maintained at 37 °C in a humidified incubator with 5% CO_2_. Three hours after seeding, the culture medium was completely replaced with maintenance medium consisting of MEM supplemented with 10% horse serum, 30 mM glucose, and 2 mM glutamine. On days 1–2 *in vitro* (DIV1–2), cytosine arabinoside (Ara-C) was added at a final concentration of 1 μM for 24 h to specifically inhibit astrocyte proliferation while minimizing neuronal toxicity. After treatment, the medium containing Ara-C was removed, the wells were thoroughly rinsed, and fresh maintenance medium was added. Neurons were further cultured until DIV7 for subsequent experiments.

### Plasmid transfection

The StayGold empty vector (pCMV-C-StayGold) (Wang et al., 2016) and the PARP-1-StayGold fusion protein expression vector (pCMV-C-PARP-1-StayGold) were generated by Suzhou GENTLEGEN Biotechnology Co., Ltd. Via a short-fragment assembly approach, and their sequences were verified by DNA sequencing. Plasmid transfection was performed using Neofect transfection reagent according to the manufacturer’s instructions. Cells were seeded into culture plates 24 hours prior to transfection. When cell confluence reached 60%–80%, plasmid DNA and Neofect reagent were separately mixed with serum-free medium according to the recommended ratios provided by the manufacturer’s instructions. The mixtures were incubated at room temperature for 15–30 min to allow formation of transfection complexes. The complexes were then added directly to the cell culture medium, gently mixed, and cells were cultured for an additional 24–48 h before subsequent treatments according to the experimental objectives.

### Molecular docking

A preliminary molecular docking prediction was performed as described previously (Xu et al., 2022). Protein crystal structures were acquired from the RCSB Protein Data Bank (http://www.rcsb.org/), and the 3D structure of Bruceine E and Brusatol ware retrieved from the PubChem database. Compound Bruceine E and Brusatol served as the ligand, and targets were used as the receptors. Semi-flexible docking of Bruceine E and Brusatol with PARP1 protein was performed using AutoDock Vina 1.1.2 software Bruceine E (mol: 21586-90-3) 3D Conformer and Brusatol (mol: 14907-98-3) 3D Conformer, PARP1 protein (Protein Data Bank code: 7KK2) (Trott and Olson, 2010). Binding site selection criteria: the binding regions were identified using the Grid Box module in AutoDockTools. Grid box sizing criteria: the docking box was dynamically adjusted to ensure coverage of all poten tial binding conformations. All water molecules and heteroatoms were removed from the receptors using PyMOL 2.4 and AutoDock Vina 1.1.2. Charges and hydrogen atoms were then added using these programs. Ligand-receptor interactions were predicted using AutoDockTools, and binding affinities were calculated based on binding energy values. A lower binding energy indicated a stronger binding affinity between ligand and receptor. 2D interaction information were generated and analyzed with LigPlot + Software (Laskowski and Swindells, 2011). Schematic diagrams created with BioGDP.com (Jiang et al., 2025).

### Molecular dynamics simulations

Molecular dynamics (MD) simulations were performed using the GROMACS 2020.6 package to evaluate the binding stability and dynamic behavior of the Bruceine E–PARP1 complex. The initial coordinates of PARP1 were retrieved from the RSCB Protein Data Bank (PDB ID: 7KK2). The protein was modeled using the CHARMM36m force field. The 3D structure of Bruceine E was optimized, and its topological parameters were generated using CGenFF to ensure compatibility with the protein force field. The complex was immersed in a dodecahedron box of TIP3P water molecules, maintaining a minimum distance of 1.0 nm between the solute and the box boundaries. To simulate physiological conditions, Na+and Cl-ions were added to neutralize the system and achieve a final salt concentration of 0.15 M. Energy minimization was executed using the steepest descent algorithm with a convergence threshold of 1000 kJ mol-1cdot nm-1. Subsequently, the system underwent two-stage equilibration: a 100 ps NVT ensemble at 310.15 K using the V-rescale thermostat, followed by a 100 ps NPT ensemble at 1.0 bar using the C-rescale barostat. The production MD simulation was carried out for 100 ns with a time step of 2 fs. The LINCS algorithm was employed to constrain all hydrogen-containing bonds. Long-range electrostatic interactions were treated using the Particle Mesh Ewald (PME) method, while van der Waals interactions were calculated with a cutoff of 1.2 nm. The temperature and pressure were maintained at 310.15K and 1.0 bar using the V-rescale and Parrinello-Rahman algorithms, respectively. Following the simulation, periodic boundary conditions (PBC) were removed. Trajectory analysis was performed using GROMACS built-in utilities to calculate the Root Mean Square Deviation (RMSD), Root Mean Square Fluctuation (RMSF), and Radius of Gyration (Rg). Intermolecular interactions were further characterized by monitoring the number of hydrogen bonds and atomic contacts (within a 0.35 nm cutoff) between the ligand and protein. The Solvent Accessible Surface Area (SASA) of the ligand was also calculated to assess its burial depth within the catalytic pocket.

### Randomization and allocation concealment

Eight-week-old male C57BL/6 mice were randomly divided into two groups. Based on body weight. Within each group, block randomization was performed by an independent statistician who was not involved in the study, using R software (version 4.3.0) with the blockrand package. The randomization sequence was concealed using the sequentially numbered, opaque, sealed envelope (SNOSE) method. Each envelope contained a group allocation card corresponding to a unique animal number, and the envelopes were stored in a locked drawer accessible only to an independent coordinator.

### Blinding

A triple-blind design was implemented. (1) Blinding of treatment administration: Drug and vehicle solutions were prepared by a technician not involved in data collection, coded as solution A or B, and verified to be identical in appearance (color, viscosity, and volume). The operator remained blinded to group allocation throughout permanent distal middle cerebral artery occlusion (pdMCAO) model induction and the entire surgical procedure. Animals were transferred to the operating room by an assistant who had opened the sealed envelope in advance and administered the coded solution 30 min before surgery. The operator had no access to any randomization materials. (2) Blinding of outcome assessment: Brain tissue sections were processed in random order. TTC-stained sections were photographed with coded filenames (e.g., S001. tif), and infarct area was quantified by an investigator blinded to group allocation using a validated ImageJ macro with automatic thresholding. (3) Blinding of statistical analysis: Group labels were recoded as Group_X and Group_Y when the dataset was transferred. The prespecified analysis plan (independent samples t-test for the primary endpoint, supplemented by sensitivity analysis) was locked before decoding. Unblinding was performed only after all analyses were completed.

### Statistical analysis

All statistical analyses were performed using GraphPad Prism 9.0. Data are presented as mean values. For comparisons between two groups, normality was first assessed using the Normality and Lognormality Tests in Prism. Normally distributed data were analyzed using independent samples t-test, while non-normally distributed data were compared using the nonparametric Mann-Whitney U test. For multiple group comparisons, one-way ANOVA was performed, followed by Dunnett’s *post hoc* test to compare each experimental group with the control group. P values for multiple comparisons were adjusted using the Dunnett method. Differences were considered statistically significant at P < 0.05.

## Results

### Traditional Chinese medicine monomer drug library screening identifies PARthanatos inhibitors

**FIGURE 1:**
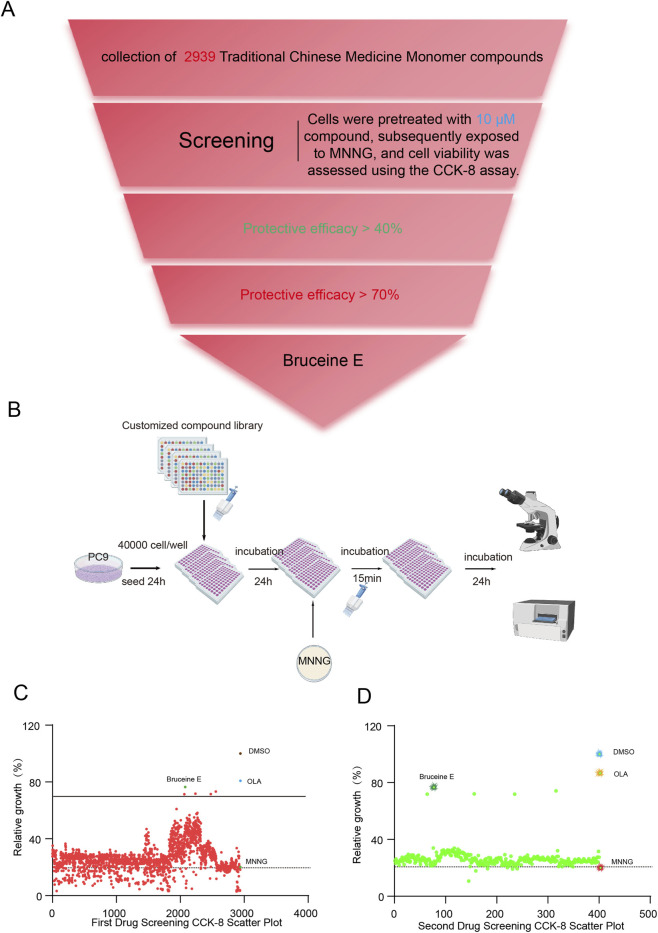
Screening of a traditional Chinese medicine monomer compound library identified candidate molecules that potently inhibit PARthanatos. **(A)** Compound library screening schematic. **(B)** Drug screening workflow schematic. **(C)** Primary screening results. CCK-8 cell viability assay showing protective effects of compounds after MNNG treatment; dashed line indicates cell viability of MNNG-treated control group. **(D)** Secondary screening validation. CCK-8 cell viability assay showing protective effects of compounds after MNNG treatment; dashed line indicates cell viability of MNNG-treated control group.

In order to identify pharmacological agents capable of mitigating the effects of MNNG (Yue et al., 2025), we conducted a screening of a traditional Chinese medicine monomer (TCM) library under conditions of N-methyl-N′-nitro-N-nitrosoguanidine (MNNG, 60 μM, 15 min) (see Supplementary Table 3). The screening strategy and process are shown in Figures 1A,B, respectively. During the initial screening phase, five compounds were identified in the library that exhibited survival rates comparable to Olaparib (OLA), a classical PARP1 inhibitor (Figure 1C). Upon rescreening, these compounds consistently demonstrated a normalized survival rate of 70% following MNNG exposure (Figure 1D). Subsequent verification revealed that the compound located in plate 26, well 8H, exhibited significant protective effects against MNNG-induced toxicity. This compound was identified as Bruceine E(BE), which demonstrated the highest survival rate and was therefore selected for further investigation.

### Bruceine E, a diterpenoid from the simaroubaceae family, is the most potent natural inhibitor of PARthanatos

Bruceine E is a quassinoid compound isolated from the seeds of Brucea javanica (L.) Merr., a species belonging to the family Simaroubaceae. B. javanica, the dried ripe fruit of this evergreen shrub, is widely distributed throughout Southeast Asia and northern Australia. Its core traditional efficacies include antimalarial activity, antidysenteric effects, heat-clearing and detoxification, and external application for wart removal. Modern pharmacological studies have confirmed its diverse biological activities, including anti-inflammatory, antimalarial, and anti-amoebic effects, which align closely with the traditional applications documented in classical medical texts (Chen et al., 2023).

**FIGURE 2:**
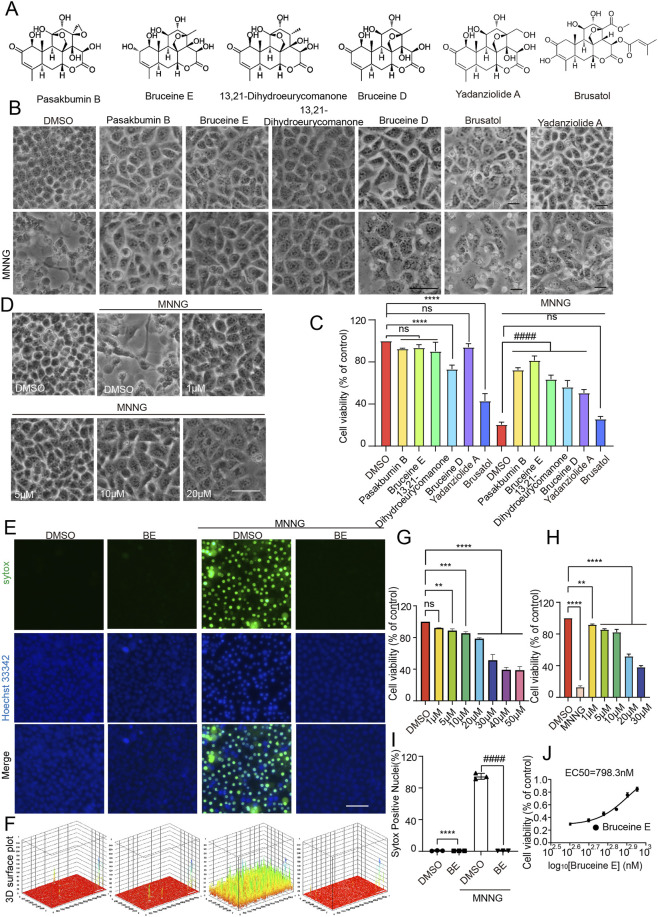
Bruceine E is the most effective quassinoid diterpenoid compound that inhibits PARthanatos. **(A)** Molecular structure of Bruceine E, Pasakbumin B, 13,21-Dihydroeurycomanone, Brusatol, Yadanziolide A and Bruceine D; **(B)** Microscopic images of cells treated with Bruceine E, Pasakbumin B, 13,21-Dihydroeurycomanone, Brusatol, Yadanziolide A and Bruceine D for 24 h, followed by MNNG treatment for 15 min and continued culture for 24 h, scale bar: 25 μm; **(C)** Cell viability was assessed using the CCK-8 assay in cells treated with Bruceine E, Pasakbumin B, 13,21-Dihydroeurycomanone, Brusatol, Yadanziolide A and Bruceine D for 24 h, followed by MNNG exposure for 15 min and continued culture for 24 h. Data are presented as mean ± SD (n = 3) (ns P > 0.05 vs. DMSO; ****P < 0.0001 vs. DMSO; ####P < 0.0001 vs. MNNG; ns P > 0.05 vs. MNNG), Statistical analysis was conducted via one-way ANOVA with Dunnett’s *post hoc* test; **(D)** microscopic images of cells treated with 1 μM, 5 μM, 10 μM, 20 μM for 24 h, treated with 60 μM MNNG for 15 min, and continued culture for 24 h in fresh medium containing Bruceine E, scale bar, 25 μm; **(E)** Typical images of cells stained with Sytox to measure cell death after MNNG treatment 18 h, scale bar: 50 μm; **(F)** 3D image of Sytox-stained green fluorescent cells measuring cell death after MNNG treatment (ImageJ-win64); **(G)** CCK-8 detection of cell viability in cells treated with Bruceine E (1 μM, 5 μM, 10 μM, 20 μM, 30 μM, 40 μM, 50 μM) for 24 h. Data represented as mean ± SD (n = 3) (ns P > 0.05 vs. DMSO; **P < 0.01 vs. DMSO; ***P < 0.001; ****P < 0.0001 vs. DMSO), Statistical analysis was conducted via one-way ANOVA with Dunnett’s *post hoc* test; **(H)** CCK-8 detection of cell viability in cells treated with 1 μM, 5 μM, 10 μM, 20 μM, 30 μM for 24 h, treated with 60 μM MNNG for 15 min, and continued culture for 24 h in fresh medium containing Bruceine E, Data represented as mean ± SD (n = 3) (**P ≤ 0.01 vs. DMSO; ****P < 0.0001 vs. DMSO), Statistical analysis was conducted via one-way ANOVA with Dunnett’s *post hoc* test; **(I)** Percentage of positive cells measured by Sytox staining after MNNG treatment. Data represented as mean ± SD (n = 3) (****P < 0.0001 vs. DMSO; ####P < 0.0001 vs. MNNG), Data distribution was evaluated by normality and log-normality tests. Independent-samples t-test was used for normally distributed data; **(J)** Curve fitting to determine the EC50 value of Bruceine E. EC50 = 798.3 nM (GraphPad Prism 10.1.1).

To elucidate the structure–activity relationship of BE in the inhibition of PARthanatos, we evaluated five additional quassinoid diterpenes with structural similarities to BE:Pasakbumin B, 13,21-Dihydroeurycomanone, Brusatol, Yadanziolide A,and Bruceine D. Their chemical structures are depicted in Figure 2A. All of these natural products possess a highly conserved C-20 skeleton, characterized by a core structure comprising three six-membered rings and a lactone ring. A distinctive structural feature is the C8–CH_2_–O–C13 oxygen bridge that spans between C-8 and C-13 within the C ring. Additionally, each of these compounds contains an α,β-unsaturated ketone, a lactone ring, and multiple hydroxyl functional groups, which collectively form the key pharmacophoric elements responsible for their biological activity. In the experimental setup, PC-9 cells were pretreated with Pasakbumin B, Bruceine E, 13,21-Dihydroeurycomanone, Brusatol, Yadanziolide A and Bruceine Dfor 24 h, followed by exposure to MNNG for 15 min to induce PARthanatos. Subsequently, the medium was replaced, and the cells were incubated for an additional 24 h. We examined morphological changes (Figure 2B) and performed CCK-8 cell viability assays (Figure 2C). Our results demonstrated that, apart from Brusatol, all five other compounds significantly inhibited MNNG-induced PARthanatos. Bruceine E exhibited the most substantial protective effect, followed by Pasakbumin B, suggesting that the quassinoid diterpene core nucleus serves as the structural foundation for PARthanatos inhibition. Furthermore, the senecioyl side chain (3-methyl-2-butenoyl) at the C-15 position of Brusatol represents the primary structural distinction from other bruceine analogues possessing PARthanatos inhibitory activity, indicating that modifications at the C-15 position may constitute the principal structural basis for target binding among bruceine derivatives. To further evaluate the safety profile of BE, cells were treated with a range of concentrationaad gradients (1 μM, 5 μM, 10 μM, 20 μM, 30 μM, 40 μM, 50 μM) for 24 h. CCK-8 cell viability assay (Figure 2G) confirmed that BE’s cytotoxicity was positively correlated with drug concentration, exhibiting a typical dose-dependent toxicity response.

To delineate the effective protective concentration range, cells were pretreated with varying concentrations of BE, specifically 400 nM, 500 nM, 600 nM, 700 nM, 800 nM, 900 nM, 1 μM, 5 μM, 10 μM, 20 μM, and 30 μM, for a duration of 24 h prior to the establishment of the MNNG injury model. Morphological assessments (Figure 2D; Supplementary Figure S1F) and CCK-8 viability assays (Figure 2H; Supplementary Figure S1E) indicated that low-to-medium concentrations of BE (1–10 μM) provided significant protection. However, as the concentration increased, intrinsic toxicity also rose, resulting in a parabolic decline in the protective effect. The half-effective concentration (EC_50_) was determined to be 798.3 nM (Figure 2J). In this study, we demonstrated that Bruceine E (BE) could effectively block MNNG-induced PARthanatos progression in human neuronal SH-SY5Y cell line and primary mouse neurons (Supplementary Figures S1A,B). Meanwhile, we further verified that BE efficiently inhibited NMDA- and glycine-triggered PARthanatos progression in primary mouse neurons (Supplementary Figures S1G-I). Consequently, a concentration of 10 μM was selected as the pharmacodynamic concentration. Sytox Green staining was employed to analyze cell death and survival (Figures 2E,F,I). The data demonstrated that BE pretreatment significantly inhibited MNNG-induced cell death, thereby confirming its potent anti-PARthanatos activity.

### BE is a PARP1 inhibitor

**FIGURE 3:**
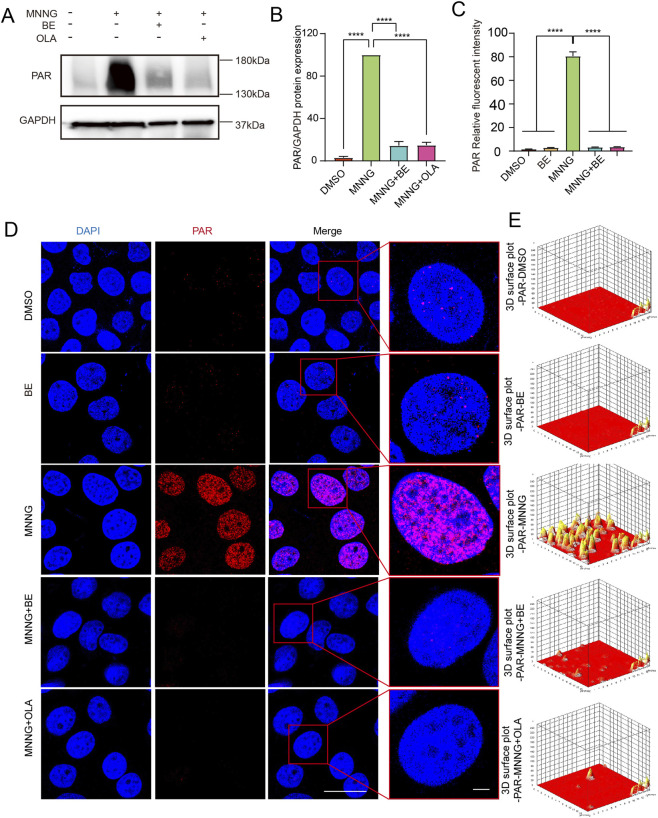
BE inhibits PARP1 activation during the progression of PARthanatos. **(A)** Immunoblotting densitometric analysis of PAR levels in PC-9 cells treated with BE for 24 h, followed by MNNG (60 μM, 15 min), and analyzed 30 min later. **(B)** Immunoblotting densitometric analysis of PAR levels in PC-9 cells treated with BE for 24 h, followed by MNNG (60 μM, 15 min), and analyzed 30 min later, Data represented as mean ± SD (n = 3) (****P < 0.0001 vs. MNNG), Statistical analysis was conducted via one-way ANOVA with Dunnett’s *post hoc* test; **(C)** Quantitative analysis of PAR fluorescence intensity in **(D)**, normalized to DAPI, Data represented as mean ± SD (n = 3) (****P < 0.0001 vs. MNNG), Statistical analysis was conducted via one-way ANOVA with Dunnett’s *post hoc* test; **(D)** PC-9 cells were pretreated with BE for 24 h, then exposed to MNNG for 15 min. Immunofluorescence staining for PAR was performed. Confocal images are shown. Scale bar: 25 μm (overview) and Magnified image, 2 μm; **(E)** 3D stereoscopic analysis image of PAR fluorescence changes by immunofluorescence, from cells pretreated with BE for 24 h and WT cells, after simultaneous treatment with MNNG for 15 min (ImageJ-win64).

A characteristic feature of early PARthanatos is increased PARP-1 activation and increased PAR modification following DNA damage. To elucidate the effect of BE on PARylation post-MNNG exposure, cells were pretreated with BE or the positive control olaparib (OLA) for 24 h, followed by a 15-min exposure to MNNG to induce PARthanatos. Cells were collected 1 h after DNA damage, and total protein was extracted for analysis. The Western blot analysis demonstrated that BE significantly inhibited MNNG-induced over-activation of PAR, with an effect comparable to that of the PARP-1 inhibitor OLA (Figures 3A,B). This finding was further corroborated at the cellular level through immunofluorescence co-localization (Figures 3C–E), which confirmed that BE effectively counteracted MNNG-induced hyper-activation of PARP1 and the subsequent increase in protein PARylation, exhibiting efficacy similar to the established PARP1 inhibitor OLA. Given BE’s substantial suppression of MNNG-induced PARylation, we hypothesized that BE may function as a novel PARP-1 inhibitor. Initial Western blot analyses of cells treated with either BE or OLA revealed no alteration in total PARP-1 protein levels (Supplementary Figures S1C,DF). Immunofluorescence imaging (Supplementary Figures S2A,B) further showed that, following pretreatment with BE or OLA, PARP-1 protein transitioned from a diffuse nuclear distribution to accumulation at sites of DNA damage. A time-course immunofluorescence assay was used to dynamically monitor the DNA damage marker γ-H2AX at 1, 6, and 18 h post-DNA injury (Supplementary Figures S2C,D). These results indicated that BE treatment did not significantly diminish the number or fluorescence intensity of γ-H2AX foci. Thus, akin to OLA, BE inhibits PARP1 activity and consequently hinders the repair of MNNG-induced DNA damage.

### BE directly binding to PARP1

**FIGURE 4:**
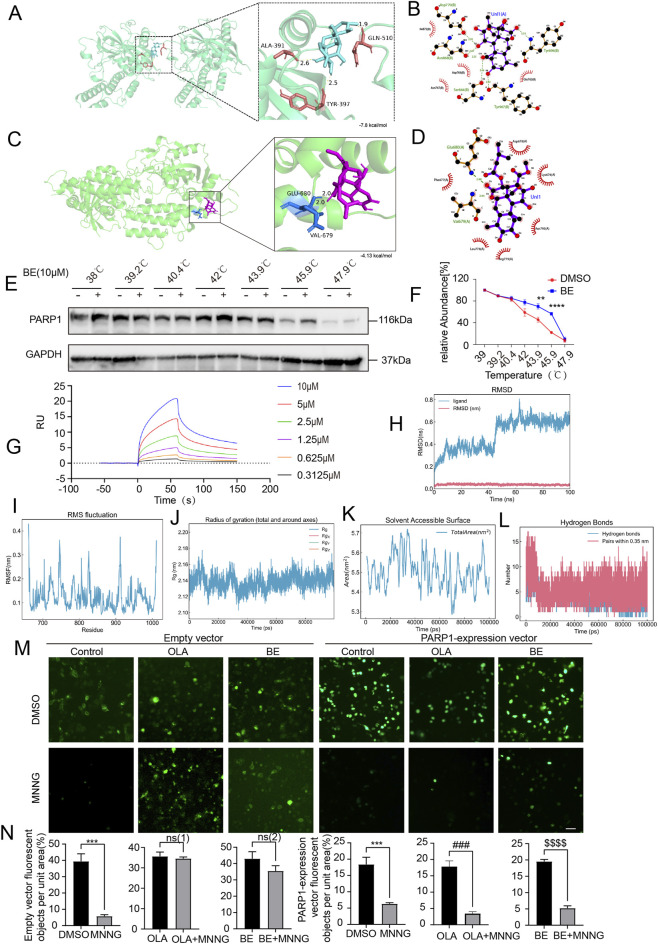
BE directly binds to the NAD^+^ catalytic pocket of PARP1. **(A)** Representative structure of BE and PARP1 after molecular docking. **(B)** LigPlot-generated relative interactions between Bruceine E and PARP1 amino acids. **(C)** Representative structure of Brusatol and PARP1 after molecular docking. **(D)** LigPlot-generated relative interactions between Brusatol and PARP1 amino acids. **(E)** Western blot detection of PARP1 thermal stability in PC-9 cells pre-treated with 10 μM Bruceine E or DMSO for 24 h (n = 3). **(F)** The intensity of Western blot bands **(C)** was quantified by densitometry with ImageJ, Data represented as mean ± SD (n = 3); (**P < 0.01 BE vs. DMSO, ****P < 0.0001 BE vs. DMSO), Data distribution was evaluated by normality and log-normality tests. Independent-samples t-test was used for normally distributed data. **(G)** Determination of KD values of BE and PARP1 protein by SPR. **(H)** Time evolution of RMSD values for PARP1 backbone and Bruceine E ligand during 100 ns molecular dynamics simulation. **(I)** Root-mean-square fluctuations (RMSF) of PARP1 residues over the 100 ns molecular dynamics simulation. **(J)** Time evolution of the radius of gyration (Rg) of PARP1 during the 100 ns molecular dynamics simulation. **(K)** Time evolution of the solvent-accessible surface area (SASA) of Bruceine E during the 100 ns molecular dynamics simulation. **(L)** Time evolution of intermolecular interactions between Bruceine E and PARP1 during the 100 ns molecular dynamics simulation. **(M)** Representative fluorescence images of cells before and after MNNG treatment in the empty vector (Vector) and PARP1-overexpressing (PARP1-OE) groups. Green fluorescence indicates viable cells, Scale bar: 35 μm; **(N)** The number of Empty Vector and PARP1-expression vector-positive cell bodies per unit area. before and after MNNG treatment in the Empty Vector and PARP1-expression vector groups. Data are presented as mean ± SD (n = 3) ***P < 0.001 vs. DMSO; ###P < 0.001 vs. OLA; $$$$P < 0.0001 vs. BE; ns (1) P > 0.05 vs. OLA; ns (2) P > 0.05 vs. BE, Data distribution was evaluated by normality and log-normality tests. Independent-samples t-test was used for normally distributed data.

To investigate the interaction between BE and PARP-1,molecular docking analysis was performed. The optimal binding conformation showed a favorable calculated binding energy of −7.8 kcal/mol (Figures 4A,B). The docking model predicts that BE binds tightly to the NAD^+^-binding catalytic pocket of PARP-1, through extensive hydrogen bonding and hydrophobic interactions. Docking studies show that BE forms six stable hydrogen bonds with PARP1: O3 (C17) with the main-chain N of Tyr896, O7 (C7) with the side-chain ND2/OD1 of Asn868, O8 (C14) with the side-chain OD1 of Asn868, and O9 (C18) with the side-chain OG of Ser864 and side-chain O of Tyr907. In this modeled pose, BE occupies the nicotinamide and ribose sub-sites of the NAD^+^ pocket, with potential to extend into the pyrophosphate region. These computational predictions suggest that BE may stabilize the D-loop of the PARP-1 catalytic domain, locking the pocket in an inactive conformation and competitively inhibiting NAD^+^ binding. Overall, the docking results support that BE is a potential PARP-1 inhibitor (Figure 4B).

Brusatol and Bruceine E are both isolated from the dried ripe fruits/seeds of *Brucea javanica* (L.) Merr., yet they exhibit distinctly different pharmacological properties. We found that, unlike Bruceine E, Brusatol fails to prevent cellular PARthanatos. Additionally, previous studies have reported that Brusatol can abrogate Nrf2-mediated cytoprotective effects, thereby promoting cell death in tumor cells and neurons in animal models of ischemic stroke (Lan et al., 2024; Zhang et al., 2025). These findings suggest that Brusatol and Bruceine E may interact with distinct molecular targets or exhibit different binding modes to the same target. We therefore further analyzed the interaction between Brusatol and PARP-1using molecular docking. The docking results revealed that Brusatol forms two weak hydrogen bonds through its hydroxyl groups at C6-O2 and C16-O3 with the backbone amide nitrogens of Val679 and Glu680, respectively. Notably, the optimal binding site of Brusatol is not located within the NAD^+^-binding pocket of PARP-1, but rather on the surface of the HD (Helical Domain) subdomain at the N-terminus of the catalytic domain (CAT, residues 656–1014). This predicted binding mode, characterized by only two hydrogen bonds, would not be expected to effectively occupy the catalytic active site. Moreover, the intermolecular interactions between Brusatol and PARP1 are fewer in number and weaker in strength, with a calculated binding free energy of −4.13 kcal/mol (Figures 4C,D).

To evaluate the binding stability of the Bruceine E–PARP1 complex, a 100 ns molecular dynamics simulation was performed. The results showed that the backbone RMSD of PARP1 underwent a conformational rearrangement at approximately 45 ns, after which it stabilized, reflecting an adaptive conformational adjustment of the protein induced by ligand binding. In contrast, the ligand Bruceine E exhibited remarkably high structural stability throughout the simulation, with its RMSD remaining around 0.05 nm (Figure 4H). RMSF analysis revealed that most PARP1 residues displayed low flexibility (<0.2 nm), with notable fluctuations observed only in a few loop regions (e.g., near residues 730, 790, 870, and 910), while the ligand-binding site remained rigid (Figure 4I). The solvent-accessible surface area (SASA) of the ligand remained stable within 5.00–5.75 nm^2^ (Figure 4K), and the radius of gyration (Rg) of PARP1 was maintained between 2.10 and 2.18 nm, confirming sustained hydrophobic burial and structural compactness of the complex (Figure 4J). Furthermore, Bruceine E formed an average of 1.9 hydrogen bonds and 12.5 atomic contacts (<0.35 nm) with PARP1 (Figure 4L). Notably, both types of interactions increased after the conformational adjustment at 45 ns, exhibiting a typical induced-fit optimization process. Collectively, these findings demonstrate that Bruceine E binds stably to the catalytic pocket of PARP1 through robust intermolecular interactions. An assessment of thermal stability in PC-9 cell lysates pre-incubated with BE or DMSO for 24 h confirmed that Bruceine E binds to PARP-1, enhancing its thermal stability (Figures 4E,F). The binding affinity between the test molecule and human PARP-1 protein was quantified using Biacore, with the protein immobilized on a CM5 chip (Figure 4G). To further verify whether the neuroprotective effect of Bruceine E was specifically dependent on the regulation of PARP1, PARP1 overexpression rescue experiments were performed. Cellular fluorescence intensity was compared before and after MNNG treatment to evaluate neuronal injury (Figures 4M,N). The experimental results demonstrated that the PARP-1 overexpression vector markedly reversed the protective effect of Bruceine E compared with the Empty Vector control group. Collectively, these findings confirm that Bruceine E exerts its neuroprotective function by inhibiting the activity and function of PARP1, thereby verifying the essential role of PARP1 in mediating the pharmacological effect of Bruceine E.

### BE protecting mitochondrial function and preventing AIF nuclear translocation

To further elucidate the molecular mechanism by which BE inhibits MNNG-induced PARthanatos, the JC-1 fluorescence probe was employed to evaluate changes in mitochondrial membrane potential (ΔΨm) 18 h post-DNA damage (Supplementary Figure S3A). The findings demonstrated that MNNG exposure led to a significant collapse of ΔΨm, whereas intervention with BE significantly mitigated this effect. Furthermore, immunofluorescence confocal microscopy was used to detect AIF nuclear translocation 18 h after MNNG treatment (Supplementary Figures S3B,C), These results demonstrated that BE effectively blocks the translocation of AIF from the mitochondria to the nucleus. Taken together, BE exerts anti-PARthanatos effects by preserving mitochondrial functional integrity and blocking AIF-mediated nuclear signaling.

### BE effectively inhibit neuron loss and affect PARthanatos-related proteins expression in mice model of permanent cerebral ischemia

**FIGURE 5:**
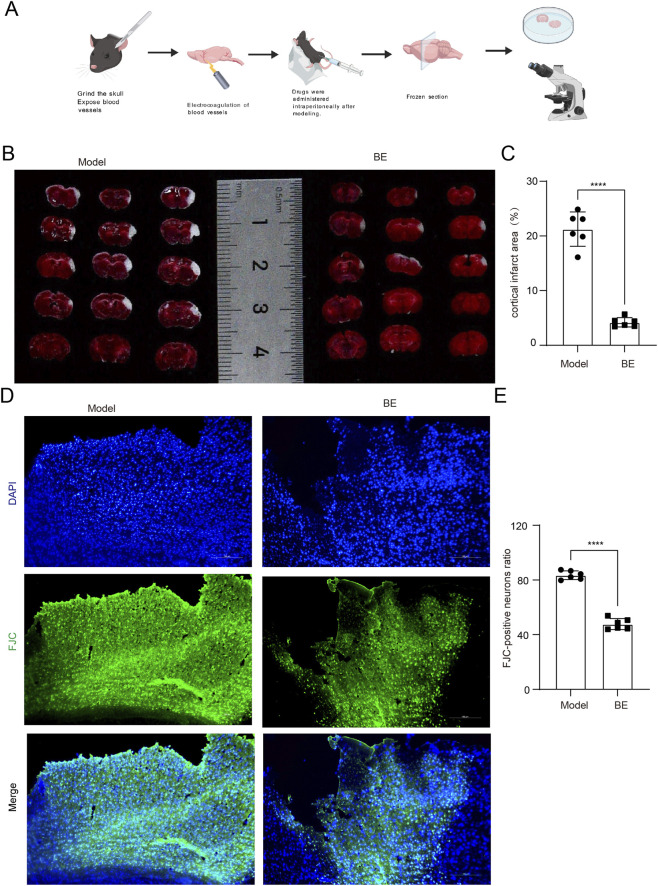
In mice model of permanent cerebral ischemia, administration of BE 4.5 h after ischemia significantly inhibits neuronal death. **(A)** Electrocoagulation ischemic stroke model flowchart. **(B)** Representative images of cerebral infarction after TTC staining at 24 h, following administration of Bruceine E (BE) at 4.5 h after modeling. **(C)** TTC staining, statistical graph of ischemic area as a proportion of hemisphere area, Data represented as mean ± SD (n = 6) (****P < 0.0001 vs. Model), Data distribution was evaluated by normality and log-normality tests. Independent-samples t-test was used for normally distributed data, Effect sizes: TTC: Cohen’s d = 7.51 (95% CI: 3.98–11.04); **(D)** FJC staining labeling images at 24 h, following administration of Bruceine E at 4.5 h after modeling (blue fluorescence indicates nucleus, green fluorescence indicates FJC-positive degenerating neurons; scale bar, 300 μm; **(E)** Statistical analysis of the rate of FJC-positive neurons, Data represented as mean ± SD (n = 6) (****P < 0.0001 vs. Model), Data distribution was evaluated by normality and log-normality tests. Independent-samples t-test was used for normally distributed data, Effect sizes: FJC: Cohen’s d = 9.71 (95% CI: 5.98–13.44).

To systematically assess the protective effects of BE against cerebral ischemic injury, we developed a mouse model of permanent ischemia through the electrocoagulation of the distal middle cerebral artery (Figure 5A). The treatment cohort received a single intraperitoneal injection of BE at a dosage of 10 mg/kg, administered 4.5 h post-occlusion. Twenty-four hours following occlusion, infarct areas were evaluated using 2,3,5-triphenyltetrazolium chloride (TTC) staining (Figures 5B,C). The findings indicated that the BE-treated group exhibited a significant reduction in cerebral infarct size compared to the model group, suggesting a strong protective effect during the acute phase. To evaluate the impact of ischemic injury on cortical neurons, Fluoro-Jade C (FJC) staining was employed to identify degenerating neurons (Figures 5D,E). The results demonstrated that ischemic injury induced substantial neuronal damage, as evidenced by an increased number of FJC-positive cells. However, in the BE-treated group, neuronal death in the ischemic region was significantly diminished, indicating pronounced neuroprotective effects.

**FIGURE 6:**
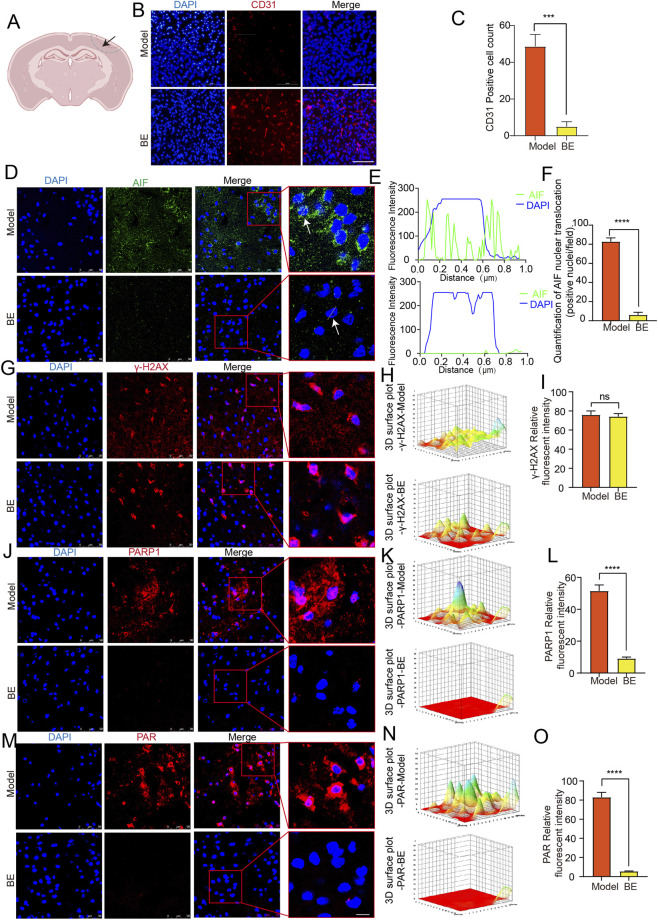
Effects of BE administration 4.5 h after ischemia on PARthanatos-related proteins in mice model of permanent cerebral ischemia. **(A)** Schematic diagram of protein extraction sites and immunofluorescence detection sites on mouse brain sections. **(B)** At 24 h after ischemia, coronal frozen brain sections from mice were subjected to immunofluorescence detection of CD31 changes. Scale bar: 200 μm; **(C)** Statistical analysis of CD31-positive microvessel numbers by immunofluorescence on frozen sections at 24 h post-ischemia,Data represented as mean ± SD (n = 3) (***P < 0.001 vs. Model), Data distribution was evaluated by normality and log-normality tests. Independent-samples t-test was used for normally distributed data; **(D)** At 24 h after ischemia, coronal frozen brain sections from mice were subjected to immunofluorescence detection of AIF changes. **(E)** Fluorescence analysis of AIF translocation by immunofluorescence on coronal frozen brain sections at 24 h post-ischemia (ImageJ-win64). **(F)** Statistical analysis of the number of AIF nuclear translocation events by immunofluorescence on frozen sections at 24 h post-ischemia, Data represented as mean ± SD (n = 3) (****P < 0.0001 vs. Model), Data distribution was evaluated by normality and log-normality tests. Independent-samples t-test was used for normally distributed data; **(G)** At 24 h after ischemia, coronal frozen brain sections from mice were subjected to immunofluorescence detection of γ-H2AX changes. Confocal images; **(H)** 3D analysis image of γ-H2AX changes by immunofluorescence from **(G)** (ImageJ-win64). **(I)** Statistical analysis of fluorescence intensity values of γ-H2AX changes from **(G)**, showing mean fluorescence intensity of γ-H2AX/mean fluorescence intensity of DAPI by immunofluorescence Data represented as mean ± SD (n = 3), (ns P > 0.05 vs. Model), Data distribution was evaluated by normality and log-normality tests. Independent-samples t-test was used for normally distributed data; **(J)** At 24 h after ischemia, following perfusion and tissue collection, coronal frozen brain sections from mice were subjected to immunofluorescence detection of PARP1 changes. Confocal images; **(K)** 3D analysis image of PARP1 changes by immunofluorescence from **(J)** (ImageJ-win64). **(L)** Statistical analysis of fluorescence intensity values of PARP1 changes, showing mean fluorescence intensity of PARP1/mean fluorescence intensity of DAPI, Data represented as mean ± SD (n = 3) (****P < 0.0001 vs. Model), Data distribution was evaluated by normality and log-normality tests. Independent-samples t-test was used for normally distributed data; **(M)** At 24 h after ischemia, following perfusion and tissue collection, frozen sections were subjected to immunofluorescence detection of PAR changes. Confocal images; scale bar: 50 μm (overview) and 25 μm (magnified detail); **(N)** 3D analysis image of PAR changes by immunofluorescence (ImageJ-win64). **(O)** Statistical analysis of fluorescence intensity values of PAR from **(M)**, Data represented as mean ± SD (n = 3) (****P < 0.0001 vs. Model), Data distribution was evaluated by normality and log-normality tests. Independent-samples t-test was used for normally distributed data.

To further investigate alterations in PARP1 and PARthanatos-related proteins following BE treatment in the permanent ischemia model, brain sections from identical anatomical locations (indicated by arrows in Figure 6A) were analyzed. Twenty-four hours post-occlusion, mice underwent perfusion, and brain sections were analyzed. Our findings revealed a significant elevation in the expression of the endothelial marker CD31 in the BE-treated group compared to the vehicle ischemia group (Figures 6B,C). This suggests that BE may protect cerebral microvascular endothelial cells from death, potentially facilitating collateral circulation or vascular remodeling and contributing to the restoration of blood flow to the ischemic region. Confocal imaging demonstrated that, in the vehicle group, apoptosis-inducing factor (AIF) translocated to the nucleus within the ischemic area, whereas BE treatment inhibited AIF nuclear import, thereby confirming the blockade of AIF-mediated PARthanatos (Figures 6E,F). The marker for DNA double-strand breaks, γ-H2AX, remained similarly elevated in both groups (Figures 6G–I), indicating that BE does not mitigate neuronal DNA double-strand breaks. However, the fluorescence intensity of poly(ADP-ribose) (PAR) in the injured area was reduced following BE treatment (Figures 6M–O), and PARP1 intensity decreased with accumulation at damage foci (Figures 6J–L), corroborating *in vitro* data. These results suggest that BE penetrates the blood-brain barrier and inhibits PARthanatos at the lesion site. In summary, BE inhibits PARP-1 activity shortly after cerebral ischemia, suppresses protein PARylation, prevents AIF nuclear translocation, and consequently blocks PARthanatos. By promoting endothelial and neuronal survival,BE provides multi-target protection that may support subsequent neurological recovery.

## Discussion

Ischemic stroke continues to lack evidence-based neuroprotective agents, primarily because previous therapeutic targets, such as calcium channels, NMDA receptors, and free radicals, are situated predominantly at “upstream” points within the ischemic cascade. Consequently, by the time pharmacological agents reach the affected area, the process of cell death has often already commenced and surpassed the “irreversible” threshold. Once the PARthanatos pathway is activated, inhibitors targeting alternative cell death pathways are ineffective (Yue et al., 2025). Therefore, the identification of novel inhibitors of the PARthanatos pathway may hold the key to achieving neuroprotection in ischemic stroke. Although clinical PARP inhibitors like olaparib and veliparib are well-established in oncology, they have not been incorporated into stroke treatment protocols due to inadequate central nervous system penetration and significant hematologic toxicity (Lin et al., 2014). By screening a traditional Chinese medicine (TCM) monomer library, we attempted to bypass the structural scaffold limitations of conventional synthetic PARP inhibitors. Using natural products as exploratory chemical probes, we identified the quassinoid diterpene Bruceine E (BE) as a potential PARP-1 inhibitor. Notably, BE possesses a distinctive tetracyclic scaffold that differs structurally from currently reported PARP inhibitors, providing alternative structural resources for developing novel PARP1 modulators. The known PARP inhibitor scaffolds include: (1) the nicotinamide core, as seen in olaparib and veliparib; (2) the naphthyridinone core, exemplified by AZD5305; (3) the quinoxalinone core, represented by AZD9574; and (4) the tricyclic heterocycle, as in talazoparib. (5) benzoxazinone core—rucaparib analogues (Roskoski, 2025).

BE, however, is a C-20 quassinoid (degraded triterpene) with a tetracyclic scaffold with a molecular weight of 412 Da, which is structurally distinct from the aforementioned five categories. Molecular docking studies reveal that BE forms six hydrogen bonds and is predicted to occupy all four NAD^+^ subsites and stabilizes the D-loop through interaction with Tyr907, resulting in a binding energy of −7.8 kcal/mol, which compares favorably to most documented natural products. The inherent rigidity and potential for Michael-addition reactivity of BE offer a chemically optimizable scaffold for the development of next-generation nM–pM PARP-1 inhibitors with improved potency. Furthermore, a 100 ns molecular dynamics simulation shows that the BE-PARP-1 complex remains structurally compact and stable throughout the simulation, with evidence of an induced-fit optimization that strengthens intermolecular interactions within the catalytic pocket. However, whether BE can be optimized to achieve nanomolar or picomolar affinity requires further medicinal chemistry efforts. Surface plasmon resonance (SPR) analysis indicates fast-on/slow-off binding kinetics, with an apparent dissociation constant (KD) of approximately 8.43 × 10^-6^ M, and a dissociation-phase half-life exceeding 30 min. Such binding characteristics indicate reversible but relatively transient binding between BE and PARP-1. This unique kinetic property enables BE to achieve rapid target engagement without irreversible covalent modification, which may partially explain the acute neuroprotective potency of single-dose BE administration. Further functional validation using PARP1 overexpression rescue experiments demonstrated that PARP1 overexpression markedly reversed the neuroprotective effect of BE compared to the empty vector control group, confirming that the pharmacological action of BE is specifically dependent on PARP1 regulation. Further long-term validation is still required to confirm its sustained therapeutic advantages and biosafety.

Brusatol exhibits weak binding affinity for PARP1, rendering it ineffective in blocking PARthanatos; moreover, it compromises the brain’s key defense mechanisms against ischemic injury by inhibiting Nrf2, thereby playing a detrimental role in the pathophysiology of ischemic stroke. These findings demonstrate that the senecioyl side chain (3-methyl-2-butenoyl) at the C-15 position of brusatol constitutes the structural basis for its lack of PARP1 binding, providing structure-activity relationship insights for future structural modifications aimed at discovering more potent PARP1 inhibitors.

In both PC-9 and SH-SY5Y human cell lines, BE effectively inhibited MNNG-induced PAR accumulation, mitochondrial membrane potential collapse, and Sytox-positive cell death, with an EC_50_ of 798 nM. This efficacy is comparable to that of olaparib, but BE exhibits a broader therapeutic window, being effective at concentrations between 1 and 10 μM and demonstrating toxicity only at concentrations exceeding 30 μM. Primary mouse cortical neurons were isolated and cultured as described previously (Wang et al., 2016). Following PARthanatos induction by glycine/NMDA, BE treatment effectively blocked this process. In a murine model of permanent distal middle cerebral artery occlusion, a single intraperitoneal injection of 10 mg/kg administered 4.5 h post-ischemia resulted in a 80.2% reduction in infarct volume. Indicating a potent neuroprotective effect not previously documented. Immunofluorescence analysis of brain sections revealed that BE inhibited AIF nuclear translocation and reduced PAR levels without affecting γ-H2AX DNA damage foci, mirroring its *in vitro* mechanism of action. Additionally, there was a significant increase in CD31^+^ micro-vessel density, suggesting that BE may also preserve vascular endothelium, thereby creating a “tissue reserve” that could facilitate later recanalization or collateral circulation. BE is classified as a quassinoid, with prior studies reporting its anti-non-alcoholic steatohepatitis and anti-diabetic properties, and an acute LD50 in mice of 31.86 mg/kg (Man et al., 2025). We observed a dose-dependent cytotoxicity at concentrations exceeding 30 μM, while no cell death occurred within the 1–10 μM range, suggesting that the observed toxicity is attributable to off-target effects and that the therapeutic window is sufficiently broad for clinical application. In contrast to the PSD-95-interfering peptide nerinetide (Hill et al., 2020), which failed in the ESCAPE-NA1 phase III trial, BE demonstrates efficacy without the need for reperfusion, potentially benefiting the 80% of patients who do not undergo thrombectomy. Unlike the NMDA antagonist aptiganel (Dyker et al., 1999), BE does not interfere with synaptic transmission and is devoid of sedative or cardiovascular side effects, rendering it a suitable candidate for use in conjunction with thrombolysis or thrombectomy.

Although early administration of PARP inhibitors has been shown to effectively inhibit cell death in renal ischemia, the concurrent inhibition of PARP-1-mediated DNA repair can lead to cellular senescence (Nehme et al., 2024). Therefore, we propose that BE could be administered during the initial 2 days following cerebral ischemia to effectively inhibit neuronal PARthanatos. Once the patient’s condition stabilizes, a transition to non-PARP-1 PARthanatos inhibitors, such as medroxyprogesterone and Bifendate, could be implemented to maintain neuronal protection while allowing PARP-1 to resume its DNA repair functions and promote DNA injured neuronal cells rejuvenation (Yue et al., 2025; Hu et al., 2026).

The limitations of this study include the investigation of only the 4.5-h post-ischemia administration window, leaving the efficacy of delayed administration at 6–24 h unresolved. In addition, this study only evaluated acute histopathological evidence at 24 h post-ischemia without assessing long-term neurological functional recovery. Furthermore, the mouse model of permanent ischemia does not fully replicate the pathology of human cerebral ischemia, highlighting the urgent need for studies utilizing non-human primate models of focal ischemia. The selectivity of BE towards PARP-2, PARP-3, and other homologues remains uncertain; therefore, cryo-EM determination of the high-resolution BE–PARP-1 complex structure is essential to guide the optimization of C-20 hydroxylation and lactone ring opening, thereby minimizing off-target effects.

This study not only introduces the quassinoid natural product into the stroke-intervention stage for the first time but also presents a patentable, translatable paradigm supporting the concept that “neuroprotection must be advanced to the irreversible node.” Subsequent systematic studies focusing on structure optimization, expansion of the therapeutic time window, immune safety, and clinical biomarkers discovery—may help address the current lack of evidence-based neuroprotectants for ischemic stroke. Furthermore, the strategy of targeting PARthanatos could potentially be extended to other DNA-damage-associated neurodegenerative conditions, such as Parkinson’s disease and neonatal hypoxic–ischemic encephalopathy.

## Data Availability

The datasets presented in this study can be found in online repositories. The names of the repository/repositories and accession number(s) can be found in the article/Supplementary Material.
