# Supplementary material for: Bruceine E, a natural quassinoid from Brucea javanica, inhibits PARthanatos via targeting PARP1 in ischemic stroke
Source: Front Pharmacol. 2026 Jun 22;17:1843673. doi: 10.3389/fphar.2026.1843673 (PMC13333763; doi:10.3389/fphar.2026.1843673)
Supplement: Supplementary file 1 [file Supplementaryfile1.docx]

**Supplementary data**

**Bruceine E, a natural quassinoid from Brucea javanica, inhibits PARthanatos via targeting PARP1 in ischemic stroke**


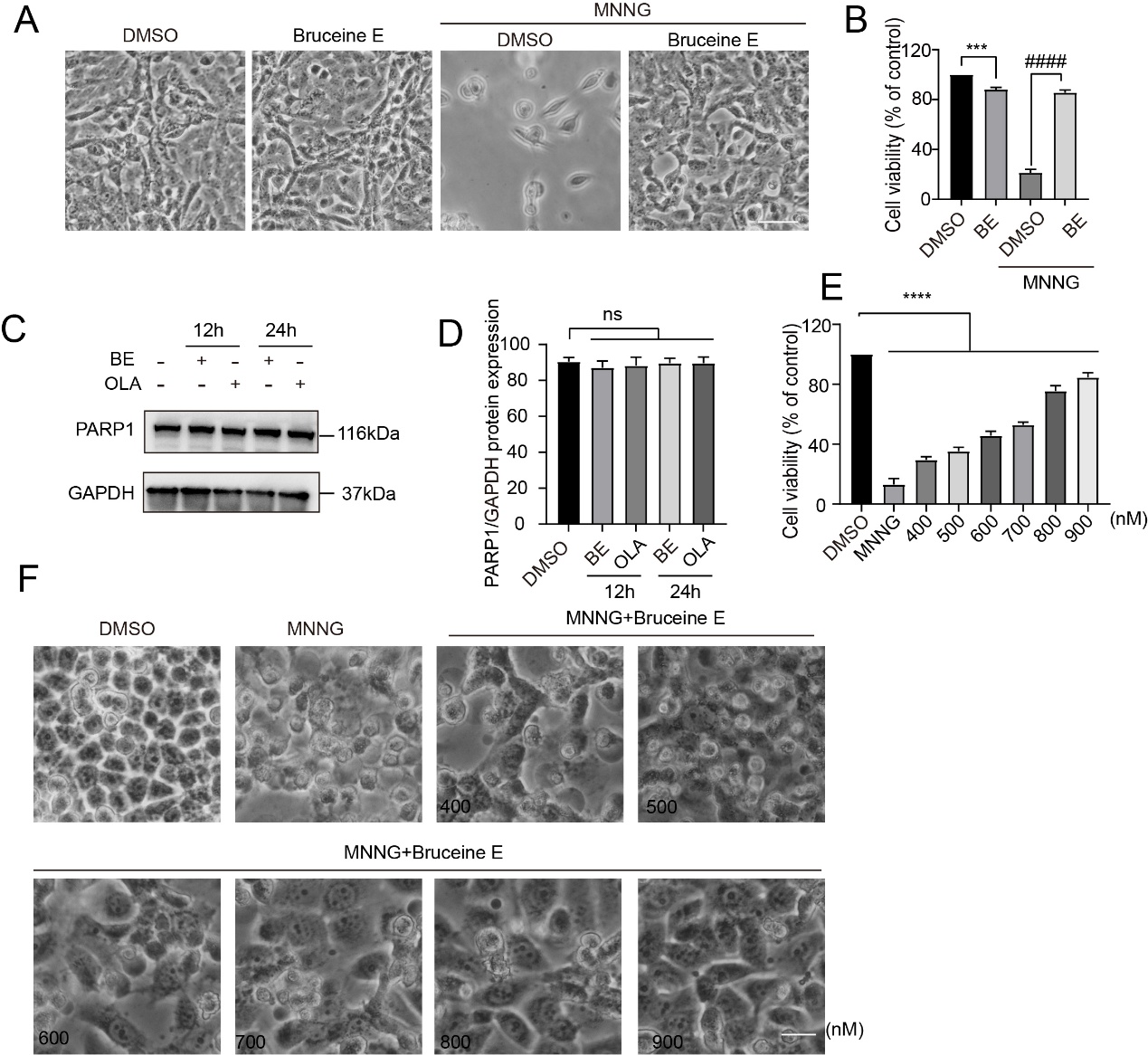


**Supplementary Figure. 1**

1. Representative phase contrast micrographs (25 μm scale bar) of SH-SY5Y cells pretreated with Bruceine E (10 μM, 24 h) followed by MNNG (60 μM, 15 min) and cultured for an additional 24 h;
2. Quantitative CCK-8 viability data (mean ± SD, n = 3; ***P < 0.001 vs DMSO; ####P < 0.0001 vs MNNG);
3. Western blot time-course (12 h and 24 h) showing total PARP-1 protein after BE treatment;
4. Densitometric quantification (PARP-1/GAPDH) confirming no significant change in total PARP-1 expression (mean ± SD, n = 3;ns P > 0.05 vs DMSO);
5. PC9 cells were treated with 400nM-900nM Bruceine E for 24 h, followed by 60 μM MNNG treatment for 15 min, then changed to fresh medium containing Bruceine E; cell viability was detected by CCK-8 assay at 24 h. Data are presented as （mean ± SD ，n = 3；****P < 0.0001 vs. DMSO).
6. PC9 cells were treated with 400nM-900nM Bruceine E for 24 h, followed by 60 μM MNNG treatment for 15 min, then changed to fresh medium containing BE; microscopy images at 24 h, scale bar, 25 μm;


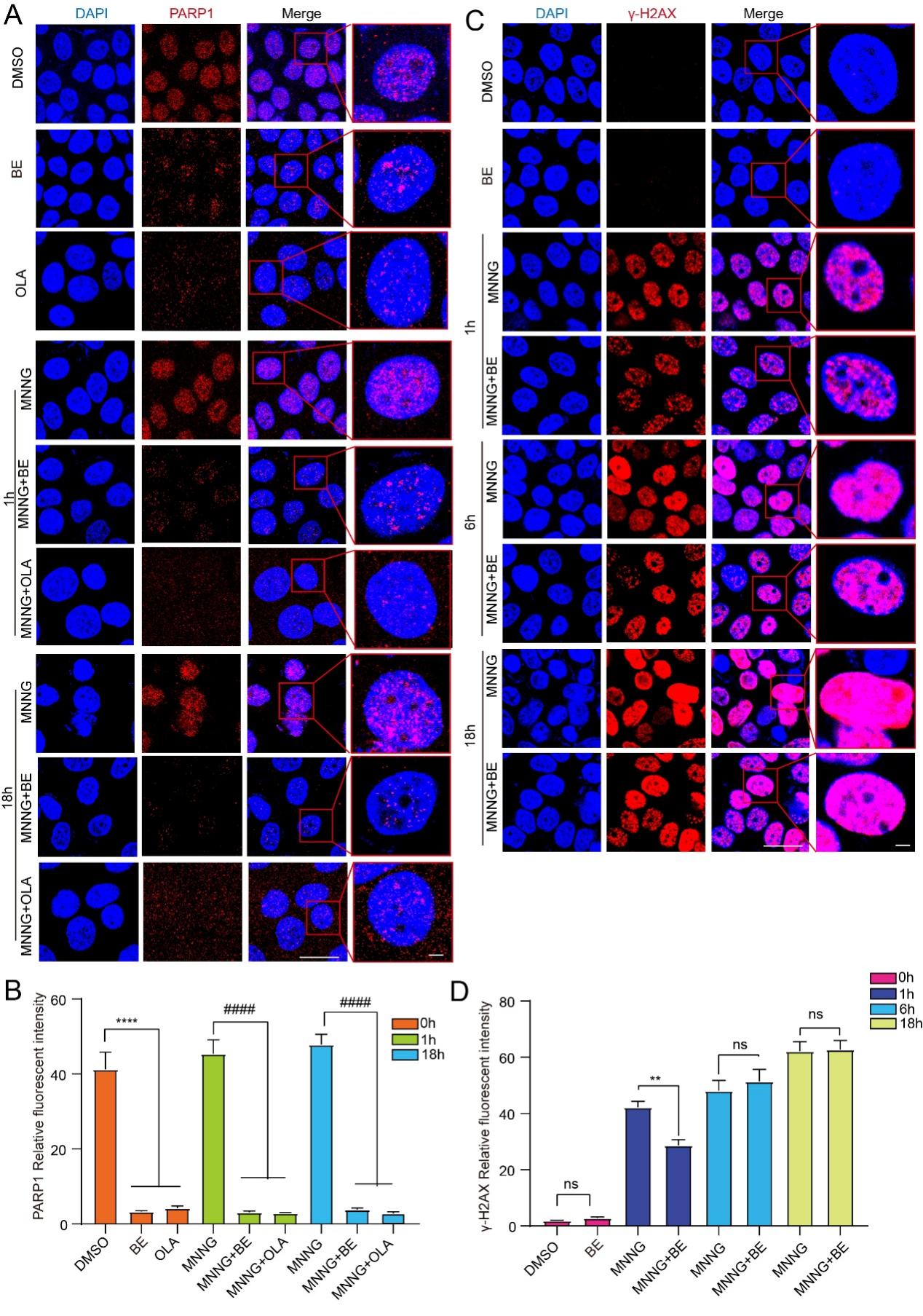


**Supplementary Figure. 2. BE does not affect PARP1 protein expression but inhibits DNA damage repair by suppressing PARP1 activity.**

1. PC9 cells were pretreated with BE for 24 hours, then exposed to MNNG for 15 minutes. Immunofluorescence staining for PARP1 was performed. Confocal images are shown. Scale bar, 25 μm, Magnified image, 5 μm;
2. Quantitative analysis of PAR fluorescence intensity in (A), normalized to DAPI, （****P<0.0001 vs.DMSO, ####P<0.0001vs. MNNG）；
3. PC9 cells were pretreated with BE for 24 hours, then exposed to MNNG for 15 minutes. Immunofluorescence staining for γ-H2AX was performed. Confocal images are shown. Scale bar, 25 μm, Magnified image, 5 μm;
4. Quantitative analysis of γ-H2AX fluorescence intensity in (C), normalized to DAPI, （ns P＞0.05 vs.DMSO, **P < 0.01 vs. MNNG, ns P＞0.05 MNNG）.


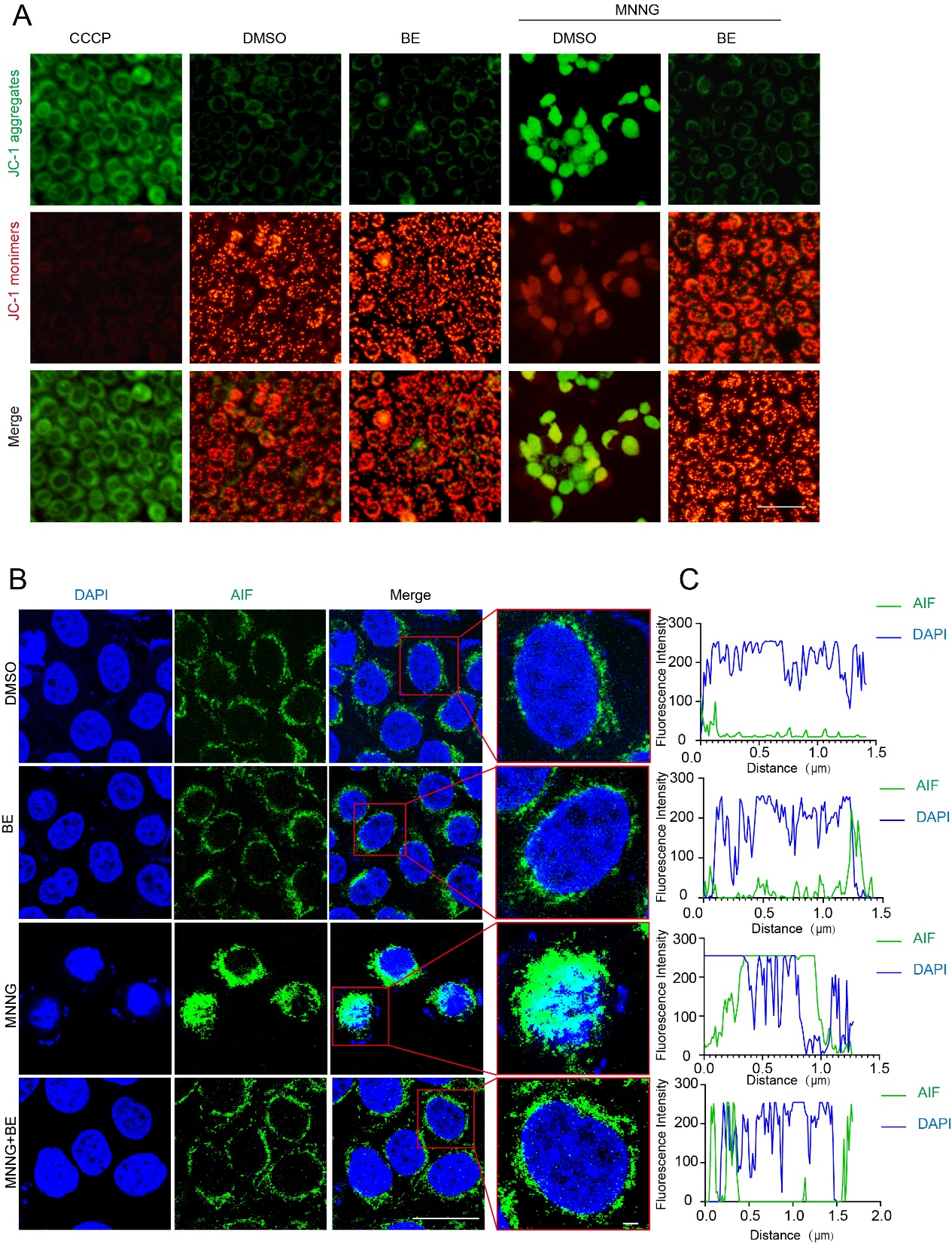


**Supplementary Figure. 3. BE pretreatment prevents MNNG-induced alteration of mitochondrial membrane potential and nuclear translocation of AIF.**

1. Typical images of JC-1-stained PC9 cells treated with BE for 24 hrs, followed by MNNG treatment for 15 minutes and continued culture for 18 hrs, scale bar, 25μm;
2. DAPI was used as a nuclear marker. Representative confocal images showing the effect of Bruceine E on MNNG-induced AIF translocation following 18 h after MNNG (60 μM, 15 min) treatment. Scale bar, 25 μm;
3. Fluorescence analysis of MNNG-induced AIF translocation.


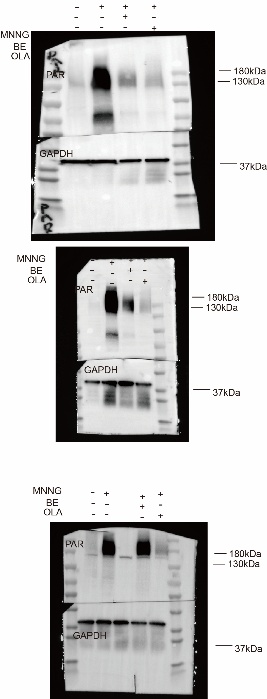


**Supplementary Figure. 4. Western Blot original images of figure 3A**


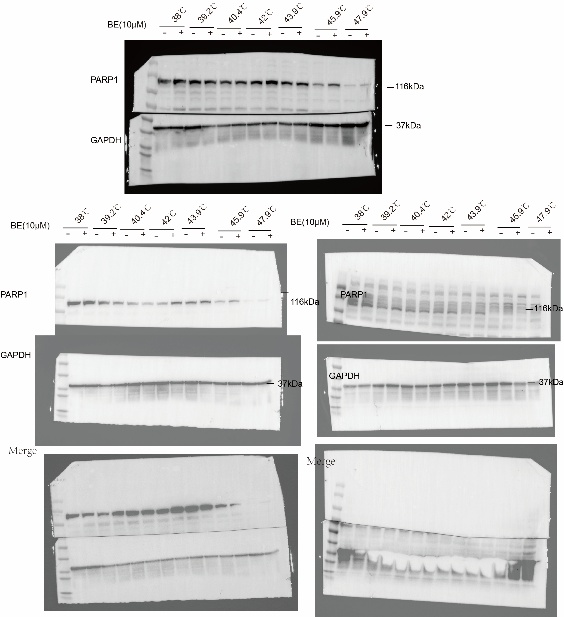


**Supplementary figure 5. Western Blot original images of figure 4E**

**
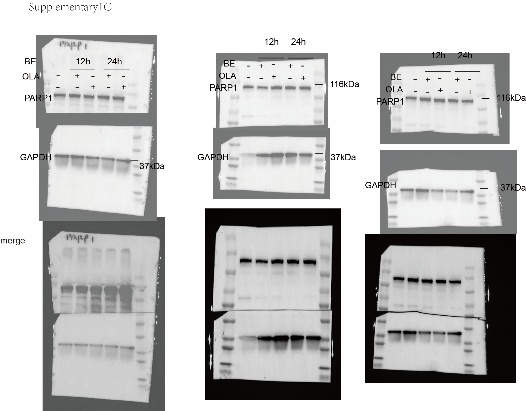
**

**Supplementary figure 6. Western Blot original images of supplementary figure. 1C**

**Supplementary Materials and methods**

**Cell culture**

Adherent cells were maintained in 10 cm dishes (Corning 430167) in complete medium: high-glucose DMEM (VivaCell, China) supplemented with 10 % FBS (VivaCell) and 1 % penicillin-streptomycin (Biosharp, China) at 37 °C in 5 % CO₂. Once cell density exceeds 90%, warm PBS, trypsin, and medium to room temperature. Remove the old medium, rinse with PBS, add trypsin, and wait for cell rounding without detachment. Remove trypsin, add fresh medium to stop digestion, create a single-cell suspension, transfer to a new dish, add medium, and incubate. Use cells at passage 3–5 with 80% confluence. For freezing, remove medium, rinse with PBS and trypsin, centrifuge at 1000 rpm for 10 minutes, discard supernatant, resuspend in freezing medium (10% DMSO + 90% FBS), aliquot 1 mL per cryovial, freeze at –80°C overnight, then store in liquid nitrogen. To thaw, rapidly warm vials in a 37°C water bath for 1–3 minutes, transfer to a centrifuge tube, add fresh medium, centrifuge at 1000 rpm for 10 minutes, discard supernatant, resuspend in fresh medium, seed into a 10 cm dish, adjust volume to 10 mL, and culture at 37°C with 5% CO₂. Check after 24 hours and change medium or passage as needed.

**Compounds treatment**

Seed cells uniformly in 12-well plates 24 h prior. At 80 % confluence add MNNG (60 μM, 15 min) to induce PARthanatos. Compounds were added 24 h before MNNG; after MNNG exposure medium was replaced with fresh compound-containing medium; 24 h later morphology was assessed under microscope.

**CCK-8 assay**

Cells were seeded at 8 × 10³ cells per well in 96-well plates (three replicates). After treatments, 10 μL CCK-8 reagent (Beyotime, China) was added per well, incubated 37 °C 2 h, absorbance read at 450 nm. Viability (%) = [(mean treated – mean blank)/(mean control – mean blank)] × 100 %.

**Protein extraction**

Pre-cool PBS and trypsin to room temperature. Remove medium, rinse with PBS, trypsin until cells round up, remove trypsin, add PBS, centrifuge 1000 rpm 4 °C 5min, discard supernatant, add RIPA lysis buffer containing 1× protease inhibitor cocktail, vortex every 5 min for 15–20 s (6 cycles), centrifuge 12 000 rpm 4 °C 15 min, collect supernatant, quantify protein, add 4× loading buffer (4:1), boil 99 °C 10 min.

**Sytox staining**

Remove medium, rinse with PBS, add medium containing Sytox Green and Hoechst 33342, incubate 37 °C 5 % CO₂ 30 min protected from light, rinse twice with PBS, image under fluorescence microscope.

**Cellular immunofluorescence**

Remove medium, rinse fixed cells with PBS 3 min × 3, permeabilized in PBT (PBS + 0.3 % Triton X-100) 30 min, block 30 min, incubate primary antibody 4 °C overnight, rinse 5 min × 3, incubate secondary antibody 2 h protected from light, rinse 5 min × 3, mount with anti-fade reagent.

**Cellular thermal shift assay**

Cells in 10 cm dishes at 80 % confluence were treated with compound or DMSO for 24 h. Cell pellets were resuspended in PBS containing 1× protease inhibitor, aliquoted into 8-tube strips (100 μL each), subjected to a temperature gradient in a PCR instrument, freeze-thawed three times (liquid nitrogen ↔ 37 °C), centrifuged 12 000 rpm 4 °C 30 min, supernatants collected for Western blot.

**Western blotting**

Equal protein (50 μg) was loaded onto SDS-PAGE gels (Wan Sheng Hao Tian, Shanghai), transferred to 0.22 μm PVDF membranes, blocked 2 h in 5 % skim milk or 1 h in protein-free blocking solution, incubated with primary antibody overnight 4 °C, washed 10 min × 3 with PBST, incubated with HRP-conjugated secondary antibody 2 h room temperature, washed 10 min × 3, visualized with ECL reagent (A:B = 1:1) using a BIO-RED imaging system. Band intensities were quantified with Imageg J software and normalized to GAPDH.

**JC-1 staining**

JC-1 reagent (Thermo Fisher, China) was equilibrated to room temperature 2–3 h before experiment. Positive-control wells were treated with CCCP (10 min), then incubated with JC-1 20 min protected from light, washed twice with PBS, imaged under consistent exposure settings. All experimental wells were processed identically.

**Animal stroke model**

Male C57BL/6J mice (7–8 weeks) from Henan SKBEX Biotechnology Co. were used. All procedures complied with ARRIVE guidelines, national regulations and were approved by the Medical Ethics Committee of Wannan Medical College (WNMC-AWE-2025489). Mice were anaesthetized with 1.5 % isoflurane, a 0.5 cm incision was made between ear and eye, fascia and muscle were blunt-dissected to expose the distal middle cerebral artery. A micro-drill thinned the skull until translucent (carefully avoiding perforation), the artery was electrocoagulated, the wound was sutured, and mice were kept warm until fully awake.

**TTC staining**

TTC solution (0.5 g TTC in 50 mL PBS) was pre-warmed to 40 °C. At 24 h post-occlusion brains were removed, rinsed in saline, frozen in a mouse brain matrix at –80 °C until firm, cut into 1 mm sections, incubated in TTC 7 min at 40 °C, flipped, incubated 4 min, fixed in 4 % paraformaldehyde 4 °C overnight, photographed the next day. Normal tissue stains red-pink; infarcted tissue remains white.

**Brain-section immunofluorescence**

Selected brain slices were washed in PBS, permeabilized in PBT (PBS + 0.3 % Triton X-100) 1.5 h at 37 °C, subjected to antigen retrieval (microwave: high 2 min, low 14 min in sodium citrate buffer), cooled, blocked 1 h at 37 °C, incubated with primary antibody 4 °C overnight, washed 7 min × 4 with PBT, incubated with fluorescent secondary antibody 2 h protected from light, washed 7 min × 4, mounted with anti-fade medium.

**Experimental Unit and Group Design**

The experimental unit was the individual mouse. Neuroprotective efficacy was assessed by comparison between model and drug treatment groups. For TTC staining determination of 24-hour infarct volume (%), experiments comprised model and drug treatment groups. Sample size was n=3 per group, with 24 mice used in total. The n=3 sample size represents the minimum scientifically justified number required to obtain reliable variance estimates while adhering to the 3R principles (Replacement, Reduction, Refinement).

**Fluoro-Jade C staining**

Brain sections were rinsed in PBS, dehydrated 5 min in 80 % ethanol + 150 μL A solution (FJC kit), 2 min in 70 % ethanol, 2 min in distilled water, incubated 10 min in 350 μL distilled water + 150 μL B solution (FJC), rinsed 2 min in distilled water, incubated 10 min in 350 μL distilled water + 150 μL C solution + 150 μL DAPI (protected from light), rinsed 1 min × 3, dried 5 min at 55 °C, then for next imaging.

**SPR methods**

Experiments were performed at 25 °C on a BIAcore T200 using CM5 sensor chips. EDC/NHS activation was followed by ligand immobilisation, ethanolamine blocking, and multi-cycle kinetics. Regeneration was with 10 mM glycine-HCl pH 2.0. Data were fitted to a 1:1 Langmuir model using Biacore Insight Evaluation software and exported to Origin 7 for final plotting. PARP1 Protein, Human (sf9, His) (MCE, Cat# HY-P74652)

**Supplementary Table 1.** List of primary and secondary antibodies

| Target protein | Company | Product code | Dilution |
| --- | --- | --- | --- |
| PAR | RD | 4335-MC-100 | 1:1000 |
| GAPDH | ABclonal | AC002 | 1:5000 |
| Goat anti-Rabbit IgG (H+L) | ABclonal | AS014 | 1:5000 |
| Goat anti-Mouse IgG (H+L) | ABclonal | AS003 | 1:5000 |
| Alexa Fluor Donkey Anti-Rabbit 488 | Jackson ImmunoResearch | 161950 | 1:200 |
| Alexa Fluor Donkey Anti-Mouse 594 | Jackson ImmunoResearch | 160927 | 1:200 |
| AIF | Santa Cruz | Sc-1316 | 1:200 |
| AIF | Cell Signaling | 4642S | 1:200 |
| CD31 | Proteintech | 66065-2-lg | 1:200 |
| PARP1 | Santa Cruz | Sc-8007 | 1:200 |
| γ-H2AX | Santa Cruz | Sc-517348 | 1:200 |

**Supplementary Table 2.** List of reagents.

| Reagent | Company | Product code |
| --- | --- | --- |
| compound library | TargetMol | A collection of 2939 Traditional Chinese Medicine Monomer compounds |
| Methylnitronitrosoguanidine(MNNG) | MCE | HY-128612 |
| Olaparib | MCE | HY-10162 |
| Bruceine E | TargetMol | 21586-90-3 |
| Pasakbumin B | TargetMol | 138809-10-6 |
| 13,21-Dihydroeurycomanone | TargetMol | 129587-06-0 |
| Yadanziolide A | TargetMol | 95258-14-3 |
| Brusatol | TargetMol | 14907-98-3 |
| Bruceine D | TargetMol | 21499-66-1 |
| Anti-fluorescence quenching sealing liquid (including DAPI) | Beyotime | P0131-25ml |
| BCA Protein Assay Kit | Biosharp | BL521A |
| RIPA Lysis Buffer | Beyotime | P0013B |
| QuickBlock™ Blocking Buffer | Beyotime | P0233 |
| Enhanced ECL chemiluminescence kit | Servicebio | G2020 |
| QuickBlock™ Blocking Buffer for Immunol Staining | Beyotime | P0260 |
| Anti-fluorescence quenching reagent | Biosharp | BL739B |
| Mitochondrial membrane potential assay kit with JC-1 | Beyotime | C2006 |
| Fluoro-Jade C | Biosensis | TR-100-FJ |
| TTC | Sangon Biotech | A610558-0025 |
| Enhanced Immunostaining Permeabilization Buffer | Beyotime | P0097 |
| QuickBlock™ Secondary Antibody Dilution Buffer for Immunofluorescence | Beyotime | P0265 |
| QuickBlock™ Primary Antibody Dilution Buffer for Immunol Staining | Beyotime | P0262 |
| DMSO | Biosharp | BL165B |
| Western and IP cell lysate | Beyotime | P0013 |
| SDS-PAGE Protein Loading Buffer (5X) | Biosharp | BL502B |
| BSA | Biosharp | BS114 |
| Antibody stripping solution (acidic) stripping buffer (low ph) | ShareBio | SB-WB007 |
| WB Protein-free Blocking Fluid (1% in 1 X TBST) Protein-free WB Blocking Buffer (TBS-T) | ShareBi | SB-PR079 |
| Fetal Bovine Serum( Superfine ) | Bio-Channel | REEBC-SE-FBSO1 |
